# Differential Inhibition of Human Atherosclerotic Plaque–Induced Platelet Activation by Dimeric GPVI-Fc and Anti-GPVI Antibodies: Functional and Imaging Studies

**DOI:** 10.1016/j.jacc.2015.03.573

**Published:** 2015-06-09

**Authors:** Janina Jamasbi, Remco T.A. Megens, Mariaelvy Bianchini, Götz Münch, Martin Ungerer, Alexander Faussner, Shachar Sherman, Adam Walker, Pankaj Goyal, Stephanie Jung, Richard Brandl, Christian Weber, Reinhard Lorenz, Richard Farndale, Natalie Elia, Wolfgang Siess

**Affiliations:** ∗ Institute for the Prevention of Cardiovascular Diseases, University of Munich, Munich, Germany; † Cardiovascular Research Institute Maastricht, Maastricht University, Maastricht, the Netherlands; ‡ advanceCOR GmbH, Munich, Germany; § Department of Life Sciences, Ben Gurion University, Beer-Sheva, Israel; ‖ GlaxoSmithKline Research & Development, Brentford, Middlesex, United Kingdom; ¶ Department of Biotechnology, The Central University of Rajasthan, Rajasthan, India; # Department of Biochemistry, University of Cambridge, Cambridge, United Kingdom; ∗∗ St. Mary's Square Institute for Vascular Surgery and Phlebology, Munich, Germany; †† DZHK (German Centre for Cardiovascular Research), partner site Munich Heart Alliance, Munich, Germany

**Keywords:** antithrombotic, atherothrombosis, glycoprotein VI, plaque rupture, ADP, adenosine diphosphate, Fc, fragment crystallizable region of IgG, GPO, glycine-proline-hydroxyproline, GPVI, glycoprotein VI, Ig, immunoglobulin, K_D_, dissociation constant, TxA_2_, thromboxane A_2_, vWF, von Willebrand factor

## Abstract

**Background:**

Glycoprotein VI (GPVI) is the essential platelet collagen receptor in atherothrombosis, but its inhibition causes only a mild bleeding tendency. Thus, targeting this receptor has selective antithrombotic potential.

**Objectives:**

This study sought to compare compounds interfering with platelet GPVI–atherosclerotic plaque interaction to improve current antiatherothrombotic therapy.

**Methods:**

Human atherosclerotic plaque–induced platelet aggregation was measured in anticoagulated blood under static and arterial flow conditions (550/s, 1,100/s, and 1,500/s). Inhibition by dimeric GPVI fragment crystallizable region of IgG (Fc) masking GPVI binding sites on collagen was compared with that of 3 anti-GPVI antibodies: BLO8-1, a human domain antibody; 5C4, a fragment antigen-binding (Fab fragment) of monoclonal rat immunoglobulin G; and m-Fab-F, a human recombinant sFab against GPVI dimers.

**Results:**

GPVI-Fc reduced plaque-triggered platelet aggregation in static blood by 51%, BLO8-1 by 88%, and 5C4 by 93%. Under arterial flow conditions, BLO8-1 and 5C4 almost completely inhibited platelet aggregation while preserving platelet adhesion on plaque. Inhibition by GPVI-Fc, even at high concentrations, was less marked but increased with shear rate. Advanced optical imaging revealed rapid persistent GPVI-Fc binding to collagen under low and high shear flow, upstream and downstream of plaque fragments. At low shear particularly, platelets adhered in plaque flow niches to GPVI-Fc–free segments of collagen fibers and recruited other platelets onto aggregates via ADP and TxA2 release.

**Conclusions:**

Anti-GPVI antibodies inhibit atherosclerotic plaque-induced platelet aggregation under static and flow conditions more effectively than GPVI-Fc. However, potent platelet inhibition by GPVI-Fc at a higher shear rate (1,500/s) suggests localized antithrombotic efficacy at denuded or fissured stenotic high-risk lesions without systemic bleeding. The compound-specific differences have relevance for clinical trials targeting GPVI-collagen interaction combined with established antiplatelet therapies in patients with spontaneous plaque rupture or intervention-associated plaque injury.

The most common cause of acute myocardial infarction and ischemic stroke is arterial thrombosis at sites of erosion or rupture of atherosclerotic plaques that expose thrombogenic plaque material to circulating blood 1, 2. We recently described a 2-step mechanism of arterial thrombus formation induced by human atherosclerotic plaques with rapid glycoprotein VI (GPVI)–mediated platelet adhesion and aggregation onto plaque collagen, followed by plaque tissue factor–mediated fibrin formation (3). Indeed, morphologically altered collagen type I and III structures present in atherosclerotic plaques 3, 4, 5, 6 are highly thrombogenic and induce platelet aggregation under static and flow conditions through binding to GPVI 3, 5, 7. In contrast to flow studies with isolated collagen fibers 8, 9, the collagen receptor α_2_β_1_ integrin is not involved in plaque-induced platelet aggregation 5, 6. Therefore, targeting GPVI might preferentially inhibit atherosclerotic plaque-induced thrombosis.

GPVI, a 60 to 65 kDa type I transmembrane glycoprotein member of the immunoglobulin (Ig) superfamily, is a main platelet collagen receptor 10, 11, 12, 13. Its expression is restricted to platelets and megakaryocytes; thus, direct targeting of this receptor does not affect other cell types (14). The monomeric form of GPVI predominates on resting platelets, but when platelets are stimulated by von Willebrand factor (vWF), collagen-related peptide, or thrombin, dimeric GPVI expression increases on the platelet surface 15, 16. Only the dimeric form of GPVI shows high affinity binding to collagen 17, 18, recognizing tandem glycine-proline-hydroxyproline (GPO) motifs in collagen fibers 9, 19. GPVI binds to collagen via its tandem Ig domains D1 and D2, which are held out from the platelet surface by an O-glycosylated mucin-like stalk (20).

GPVI deficiency causes only a limited bleeding tendency, reinforcing its potential as a selective and relatively safe drug target 10, 14, 21. The GPVI-collagen interaction can be inhibited either by occupation of GPO-binding sites on collagen using extracellular GPVI fused to the Fc region of human IgG (GPVI-Fc, Revacept, advanceCOR, Munich, Germany) or by antibodies directed against platelet GPVI. In phase I studies, GPVI-Fc was well tolerated without affecting systemic hemostasis in healthy human volunteers. It inhibited collagen-induced platelet aggregation ex vivo in a dose-dependent manner (22). A human recombinant Fab (m-Fab-F) specifically blocks GPVI dimers (18). BLO8-1, a human anti-GPVI domain antibody consisting of a single Ig variable domain recognizes residue K59 in domain D1 on the apical surface of GPVI (23). 5C4, the Fab fragment of a monoclonal GPVI-blocking rat IgG, targets epitopes of GPVI at D1 and the intersection to domain D2 (24).

The aim of this study was to explore the platelet-inhibiting potential of GPVI-Fc and anti-GPVI antibodies under both static and arterial flow conditions. Blood was stimulated with human atherosclerotic plaque material to mimic pathophysiological conditions of plaque rupture.

## Methods

Atherosclerotic plaques were obtained from patients undergoing endarterectomy for high-grade carotid artery stenosis. Patient informed consent was obtained, as approved by the Ethics Committee of the Faculty of Medicine of the University of Munich in accordance with the ethical principles for medical research involving human subjects as set out in the Declaration of Helsinki.

The carotid plaque tissue was endarterectomized, processed, and preserved as described 3, 25. Plaque homogenates from 5 patients were mixed to obtain plaque pools that were kept in aliquots at −80°C. Plaque homogenates were used for platelet aggregation studies or coated onto glass coverslips for flow studies 3, 26, 27.

We stimulated blood with plaque homogenates containing all potential thrombogenic compounds or Horm collagen. Plaque contains mainly type I and III collagens 5, 6. Horm collagen consists of collagen type I, as suggested by the supplier, but also collagen type III (W. Siess, unpublished observations, October, 2013). In addition to experiments with blood pre-incubated with GPVI-Fc and subsequent plaque stimulation, we performed experiments with plaque pre-incubated with 35-fold or 50-fold higher concentrations of GPVI-Fc than finally present in blood to maximally saturate the collagen-binding sites for GPVI.

For additional details, see the Online Appendix.

## Results

**Figure 1:**
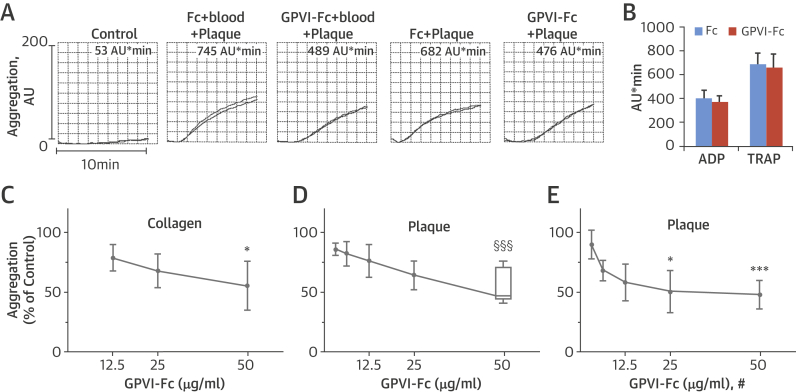
Static Platelet Aggregation Attenuated by GPVI-Fc **(A)** Representative multiple electrode aggregometry tracings show plaque-induced platelet aggregation in blood pre-incubated with solvent, equimolar concentrations of Fc (16 μg/ml), and GPVI-Fc (50 μg/ml) (tracings 1 to 3). Plaque samples pre-incubated with 35-fold higher equimolar concentrations of Fc (560 μg/ml) or GPVI-Fc (1,750 μg/ml) for 3 min were added to blood yielding the same final concentrations (tracings 4 and 5). Numbers show cumulative aggregation (AU*min) measured from 0 to 10 min. **(B)** GPVI-Fc does not affect platelet aggregation stimulated by ADP (5 μM) or TRAP (15 μM) (mean ± SD, n = 4). Blood was pre-incubated with GPVI-Fc or Fc control protein in increasing concentrations before stimulation with collagen (0.5 μg/ml) **(C)** or with plaque (833 μg/ml) **(D)** for 5 min. **(E)** Plaque was pre-incubated with equimolar concentrations of GPVI-Fc (109, 219, 437, 875, and 1,750 μg/ml) or Fc for 3 min before added to blood, yielding the same final concentrations (#) as in **D**. *p < 0.05; ***p < 0.001 for GPVI-Fc versus control by 2-tailed paired Student *t* test, or §§§p < 0.001 by the Mann–Whitney *U* test. ADP = adenosine diphosphate; Fc = fragment crystallizable region; GPVI = glycoprotein VI; TRAP = thrombin receptor-activating peptide.

Aggregation measurements showed that GPVI-Fc, but not Fc lacking the external GPVI domain, delayed plaque- and collagen-stimulated platelet aggregation in blood (Figure 1A). In plaque-stimulated samples, the lag time until the start of aggregation increased from 65 ± 19 s with Fc control protein to 119 ± 23 s (n = 6; p < 0.001) with GPVI-Fc (50 μg/ml, 300 nM), when the proteins were pre-incubated with blood and from 72 ± 20 s with Fc control protein to 134 ± 25 s (n = 6; p < 0.001) with GPVI-Fc, when the proteins were mixed with plaque before blood stimulation. Inhibition was specific because GPVI-Fc did not affect platelet aggregation when stimulated with adenosine diphosphate (ADP) and thrombin receptor-activating peptide (Figure 1B). Collagen-stimulated platelet aggregation was dose dependently reduced (maximally by −45%) (Figure 1C) as was plaque-induced platelet aggregation by blood pre-treatment with GPVI-Fc (maximally by −51%) (Figure 1D). Surprisingly, inhibition was not significantly enhanced if plaque was pre-incubated with a 35-fold higher GPVI-Fc concentration before stimulation of the blood samples (Figure 1E), even if pre-incubation time was extended to 30 min (Online Figure 1).

The anti–GPVI antibodies BLO8-1 (10 μg/ml, 833 nM) and 5C4 (1.25 μg/ml, 25 nM) almost completely inhibited plaque- and collagen-induced platelet aggregation in a concentration-dependent manner (Online Figures 2A and 2B, and not shown). The highest concentration of BLO8-1 decreased aggregation to 12% of control (n = 9) after plaque stimulation and to 16% (n = 8) after collagen stimulation. Residual aggregation after pre-incubation with the highest 5C4 concentration was 7% on plaque stimulation (n = 5) and 18% on collagen stimulation (n = 5). Inhibition was specific because neither BLO8-1 nor 5C4 affected platelet aggregation when stimulated by ADP and thrombin receptor–activating peptide (Online Figure 2C).

Because dimeric GPVI on resting platelets is essential for collagen binding and platelet activation (16), we performed experiments with m-Fab-F directed against dimeric GPVI 16, 18 and compared it with 5C4, which blocks monomeric and dimeric GPVI. The m-Fab-F inhibited plaque-induced platelet aggregation less effectively than 5C4 (−64 ± 11% vs. −86 ± 8%; p < 0.05). Inhibition of plaque-induced platelet aggregation by dimeric GPVI-Fc was −53 ± 17% (Online Figure 3). 5C4 inhibits platelet aggregation with a half maximal inhibitory concentration (IC_50_) of ∼0.2 μg/ml, corresponding to a dissociation constant (K_D_) of ∼1 nM, whereas m-Fab-F has a reported K_D_ for GPVI dimer of ∼10 nM. However, although m-Fab-F binding to GPVI dimer is saturable, lower maximal available binding sites (B_max_) were reached using m-Fab-F than using other antibodies (16), indicating that m-Fab-F does not bind to all GPVI dimers present on the platelet surface.

To simulate plaque rupture and subsequent platelet activation, human whole blood was perfused in a parallel plate flow chamber over human plaque homogenate at different arterial shear rates: 550/s and 1,100/s are within the range of physiological mean and peak wall shear rates of carotid and coronary arteries 28, 29, and shear rates of ∼1,500/s prevail over mildly stenotic coronary lesions.

The fluorescence micrographs in Figures 2A and 2B and diagrams in Figure 2C (quantifying the area covered with platelets over time) show inhibition of plaque-induced platelet deposition by GPVI-Fc, BLO8-1, and 5C4 at different arterial shear rates. Platelet coverage tested at full minutes for all treatments and shear rates of 550/s and 1,100/s and for GPVI-Fc versus control for shear rates of 550/s, 1,100/s, and 1,500/s by 3-way analysis of variance was significant for factors treatment (p < 0.001), shear (p < 0.05), time (p < 0.001), and the interaction of treatment with shear (p < 0.05) and time (p < 0.001). GPVI-Fc (50 μg/ml) significantly delayed and reduced plaque-induced platelet aggregation compared with controls (Figures 2A and 2C, Online Videos 1 and 2). Because the limited inhibition at the shear rate of 550/s might be explained by subsaturating blood concentrations of GPVI-Fc not blocking all tandem GPO motifs in plaque collagen, we pre-incubated plaque-coated coverslips with 50-fold higher GPVI-Fc concentrations (than reached after GPVI-Fc addition to blood) before low shear rate flow blood perfusion. As in the static experiments (Figure 1, Online Figure 1), inhibition by GPVI-Fc was not increased (Online Figure 4).**Figure 2:**
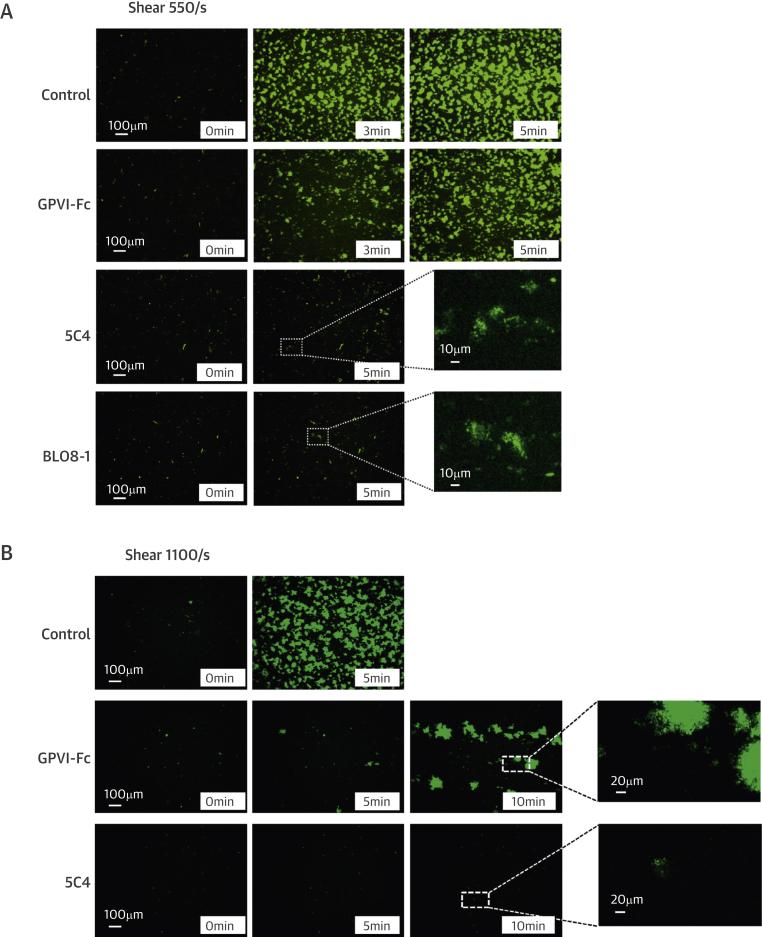

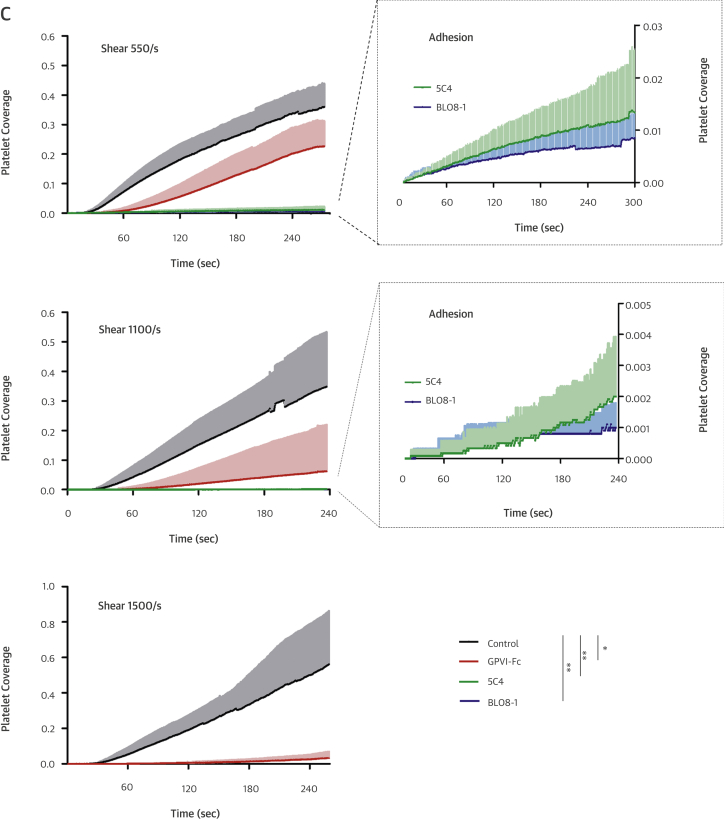
Inhibition of Atherosclerotic Plaque-Induced Platelet Deposition by GPVI-Fc and Anti-GPVI Antibodies Representative micrographs display platelet coverage of plaque at different times after start of blood flow at 550/s **(A)** (Online Videos 1 and 2) or 1,100/s **(B)**. Blood was pre-incubated with mepacrine for platelet visualization (without = control) or with GPVI-Fc (50 μg/ml) or anti-GPVI antibodies 5C4 (1.25 μg/ml) or BLO8-1 (20 μg/ml). Enlarged insets = high magnification images. **(C)** Effect of GPVI-Fc, BLO8-1, or 5C4 on the time course of platelet deposition onto plaque from flowing blood at 3 different arterial shear rates (Online Videos 1 and 2). BLO8-1 and 5C4 curves are shown at blown-up scale **(right)**. Mean ± SD of 5 to 12 experiments. Secondary pair comparisons between treatments were significant for control versus Blo8-1 (**p < 0.01), 5C4 (**), and GPVI-Fc (*p < 0.05). Abbreviations as in Figure 1.

**Figure:**
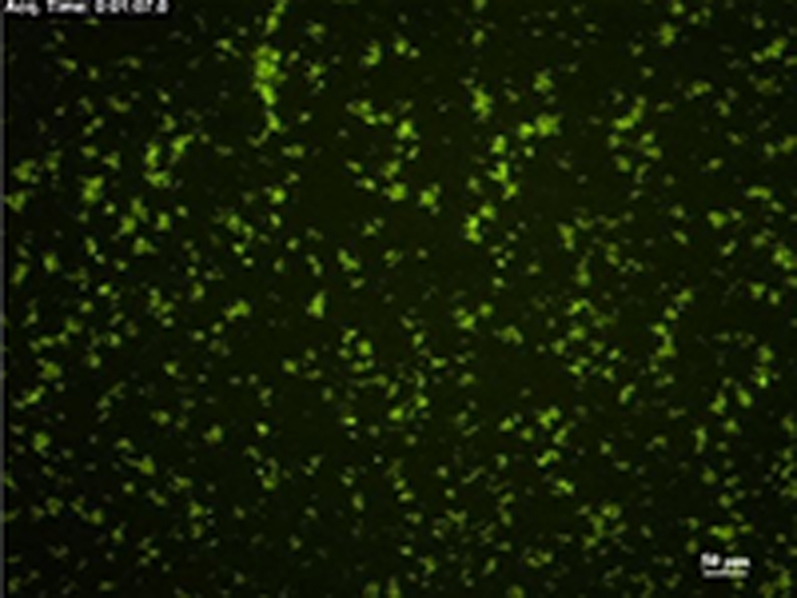
Online Video 1

**Figure:**
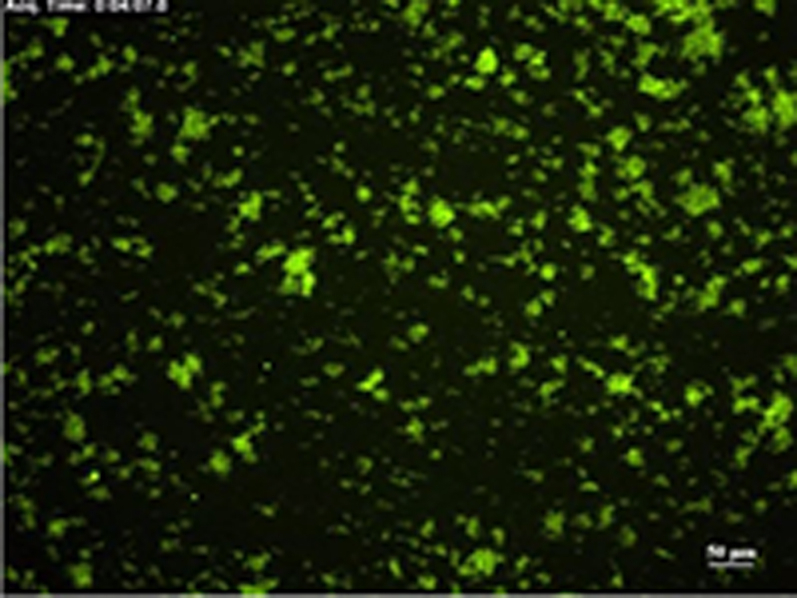
Online Video 2

**Figure:**
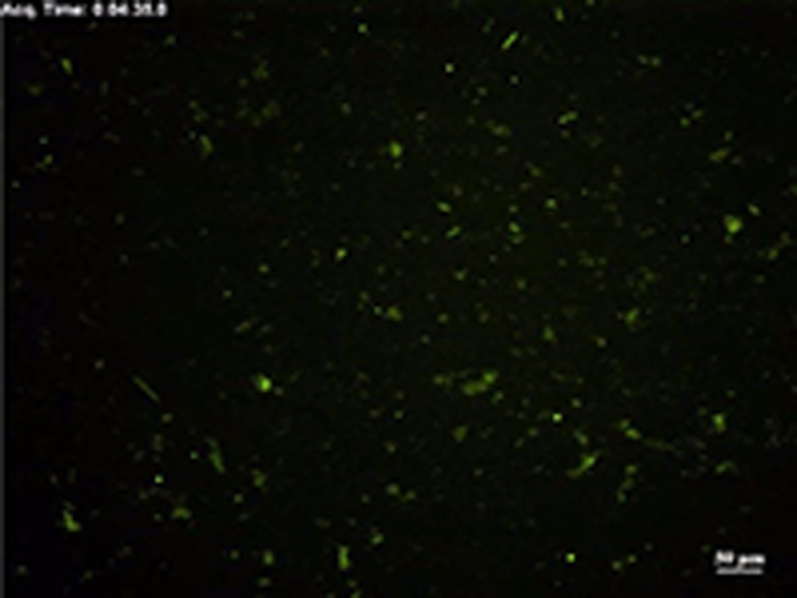
Online Video 3

Interestingly, inhibition by GPVI-Fc increased with shear rate. At 1,500/s, GPVI-Fc effectively inhibited plaque-induced platelet aggregation (Figure 2C, bottom).

The anti-GPVI antibodies BLO8-1 (20 μg/ml) and 5C4 (1.25 μg/ml) almost completely inhibited platelet aggregate formation at shear rates of 550/s and 1,100/s. Only platelet adhesion was observed, which was predominantly transient (Figure 2, Online Video 3).

### Advanced optical imaging

To study the mechanism of the GPVI-Fc–mediated inhibition of platelet aggregate formation on plaque, we visualized the binding of fluorescent GPVI-Fc to plaque in relation to platelet adhesion and aggregate formation in flowing blood. GPVI-Fc rapidly bound to plaque, reaching saturation 250 s after start of flow (Figure 3A). GPVI-Fc bound faster to plaque than platelets, but the kinetics and amount of GPVI-Fc binding were similar at low (550/s) and high (1,500/s) shear rates. This excludes a difference in GPVI-Fc binding as an explanation for the GPVI-Fc superior inhibition of platelet deposition at a high shear rate.**Figure 3:**
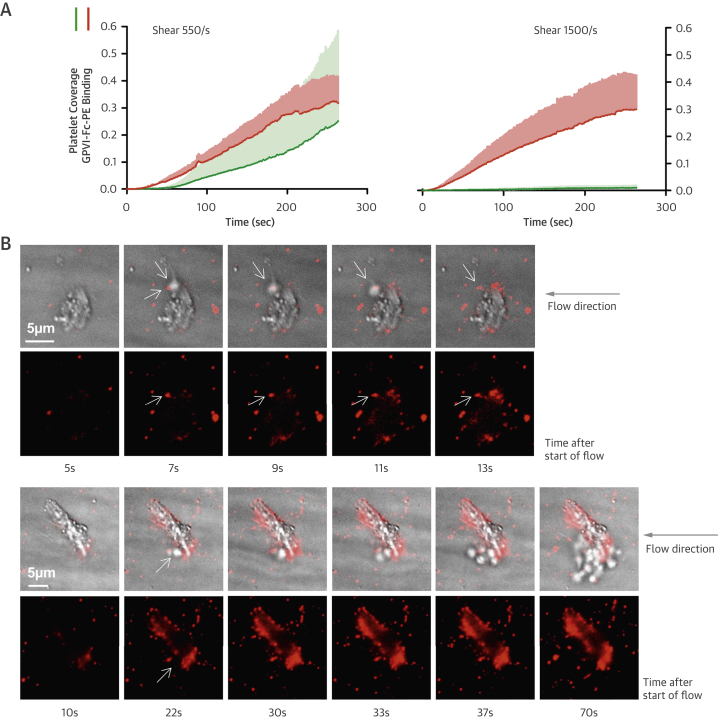
Dynamics of GPVI-Fc Binding, Platelet Adhesion, and Aggregate Formation Onto Atherosclerotic Plaque Material Under Flow **(A)** GPVI-Fc (50 μg/ml final concentration) labeled with phycoerythrin (PE)–conjugated anti-human Fc antibody **(red)** and added to blood before perfusion rapidly bound to plaque homogenate. Binding was similar at low and high shear rates (550/s and 1,500/s). Deposition of platelets (**green**; labeled with DiOC6) lagged behind at low shear and was inhibited at high shear. **(B)** GPVI-Fc-PE **(red)** binds up- and downstream of plaque fragments **(gray)** and to small plaque pieces not detectable by differential interference contrast (DIC) (Online Videos 4, 5, and 7). Platelet adhesion and aggregate formation **(gray)** is observed only downstream. Phase contrast (DIC) images of platelet **(gray)** and GPVI-Fc-PE binding **(red)** to plaque at different times after start of blood perfusion at low shear rate (550/s). **Rows 1 and 2:** A single platelet **(upper arrow)** rolls over PE-labeled GPVI-Fc **(lower arrow)** bound to a piece of plaque material. **Rows 3 and 4:** Platelet aggregate formation starting from a single adhering platelet **(arrow)** in a flow niche downstream of plaque. **Rows 1 and 3:** Overlay of DIC (plaque/platelets) and fluorescence (PE-labeled GPVI-Fc) images; rows 2 and 4: fluorescence images of PE-labeled GPVI-Fc. Bar = 5 μm. Abbreviations as in Figure 1.

**Figure:**
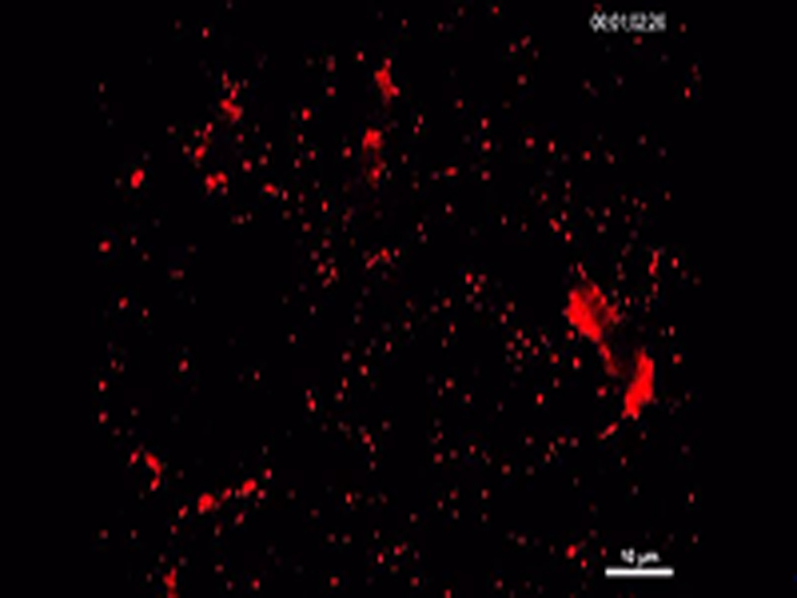
Online Video 4

**Figure:**
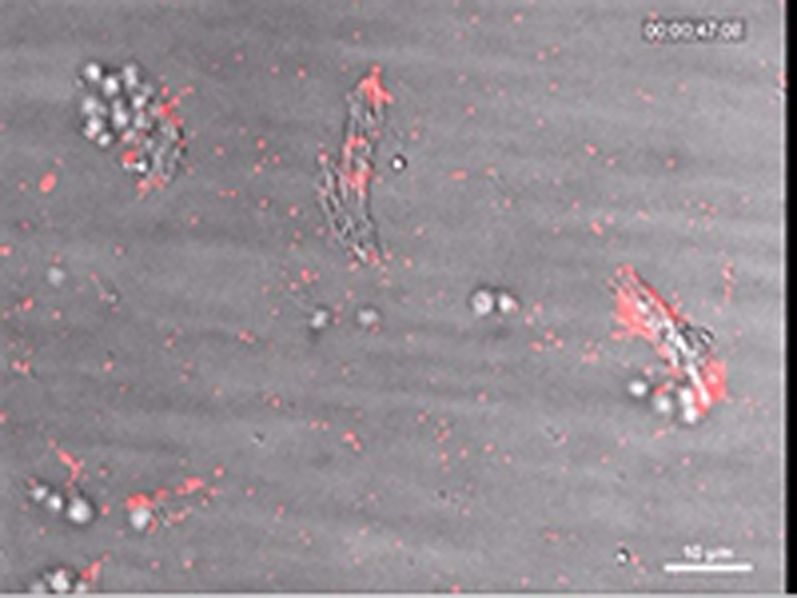
Online Video 5

**Figure:**
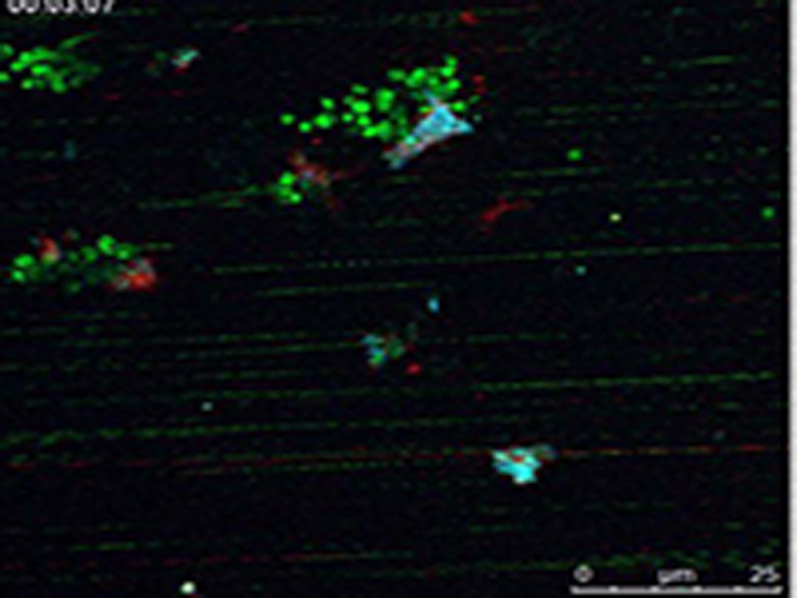
Online Video 6

**Figure:**
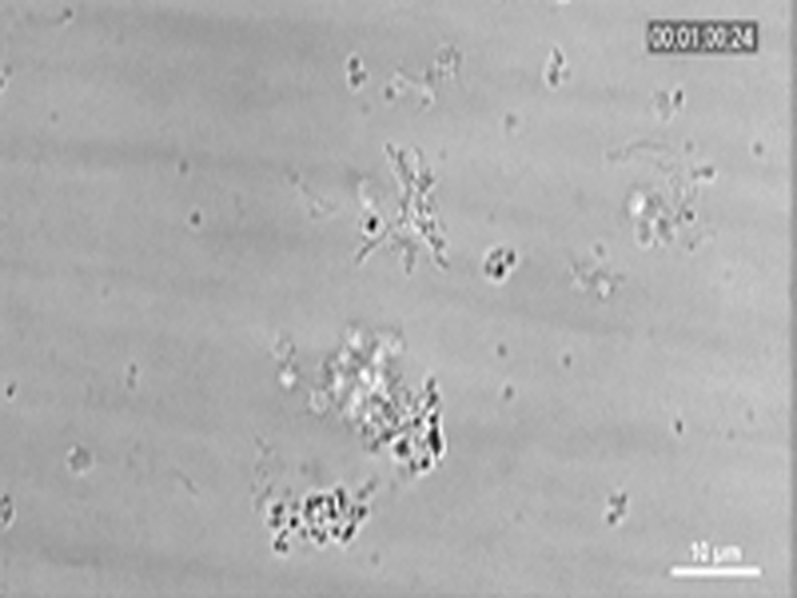
Online Video 7

We visualized GPVI-Fc binding and platelet attachment to plaque by high magnification bright-field and fluorescence video microscopy during the first minutes of flow (Figure 3B). Phycoerythrin-labeled GPVI-Fc added to blood rapidly bound as discrete dots to small plaque particles as well as to large plaque fragments up- and downstream, mainly in an irreversible manner (Figure 3B, Online Videos 4 and 5). GPVI-Fc binding to the small, more homogenized plaque components was more rapid than binding to the whole plaque homogenate containing the large plaque fragments (Figure 3A, Online Figure 5). The dots representing GPVI-Fc labeled with phycoerythrin-conjugated anti–human Fc antibody differed in size and intensity most likely reflecting the size of the fluorescent GPVI-Fc/antibody complexes. Fluorescent GPVI-Fc binding to plaque was specific. It did not bind to albumin-blocked glass surface, and fluorescence- labeled Fc protein added to blood did not bind to plaque.

Compared with GPVI-Fc binding, platelet adhesion to plaque was infrequent and mostly transient, and platelet aggregate formation onto plaque was much slower (Figure 3A, Online Video 5). Fluorescent GPVI-Fc prevented platelet attachment to plaque. As shown in Figure 3B, row 1, a single platelet was rolling over fluorescent GPVI-Fc bound to a plaque fragment. However, some platelets were able to irreversibly adhere to sites downstream of protuberant plaque fragments (Figure 3B, row 3, Online Video 5); this was more pronounced at low versus high shear rates. Such platelets then served as a starting point for platelet aggregate formation (Figure 3B, row 3).

Two-photon laser scanning microscopy showed 3-dimensional (3D) platelet aggregate formation up- and downstream of plaque material at low and high shear in untreated blood. At low and high shear, AlexaFluor594-labeled GPVI-Fc added to blood rapidly bound to auto-fluorescent small and large plaque fragments before platelet adhesion. At low shear, platelet adhesion and subsequent aggregate formation in the presence of GPVI-Fc was only observed downstream of plaque material (Online Figure 6A, Online Video 6), that was confirmed by high magnification differential interference contrast video microscopy (Figure 3B, row 3, Online Video 7). At high shear, platelet attachment downstream of the plaque was unstable in the presence of GPVI-Fc and much less platelet aggregate formation was observed (Online Figure 6B).

To better visualize the plaque sites to which platelets adhered under flow (550/s) in the presence of GPVI-Fc, we performed super-resolution microscopy using structured illumination microscopy (SIM) (30). GPVI-Fc bound as dots, often in a string-like pattern, to collagen upstream and downstream of plaque fragments, yet leaving stretches of plaque collagen unoccupied by GPVI-Fc (Figure 4, 3-dimensional image in Online Video 8). Platelets adhered to discrete sites of collagen fibers at different z-levels of plaque. These sites were sometimes in close proximity to GPVI-Fc binding of collagen. Also in the structured illumination microscopy studies, platelets only adhered downstream of plaque fragments.**Figure 4:**
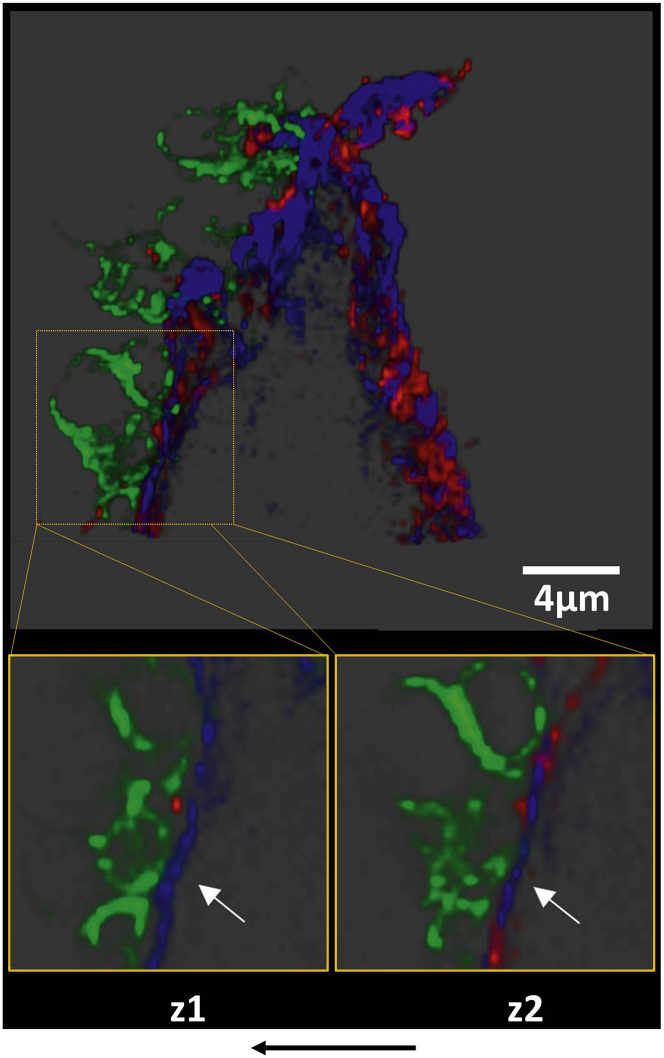
Platelets Adhere Downstream to Sites of Plaque Collagen Not Occupied by But in Close Proximity to GPVI-Fc as Revealed by SIM Imaging Plaque homogenates pre-stained with anti-collagen types I and III antibody (Ab) and Alexa Fluor 405 conjugated second Ab were perfused with blood containing Alexa Fluor 594–labeled GPVI-Fc **(red)** (50 μg/ml) and abciximab (to block platelet aggregation) at a shear rate of 550/s. After 3 min of flow, samples were fixed, and platelets **(green)** were stained with anti-CD41 Ab and DyLight 488 conjugated second Ab. Structured illumination microscopy fluorescence micrographs were taken of the subsequent 0.2-μm sections of the sample, and 3-dimensional reconstructions were made with ImagePro Premier 3D (version 9.1, Media Cybernetics, Rockville, Maryland). **(Top)** Three-dimensional overview of the sample (thickness, 3.6 μm). **(Bottom)** Magnified subvolumes of the sample at 2 z positions from bottom to top (z1 = 1.0 to 1.6 μm; z2 = 1.6 to 2.2 μm) revealing platelet adhesion to discrete sites of plaque collagen **(blue, arrows)**. **Black arrow** shows direction of blood flow. Image is representative of 7 others (see also Online Video 8). SIM = structured illumination microscopy.

**Figure:**
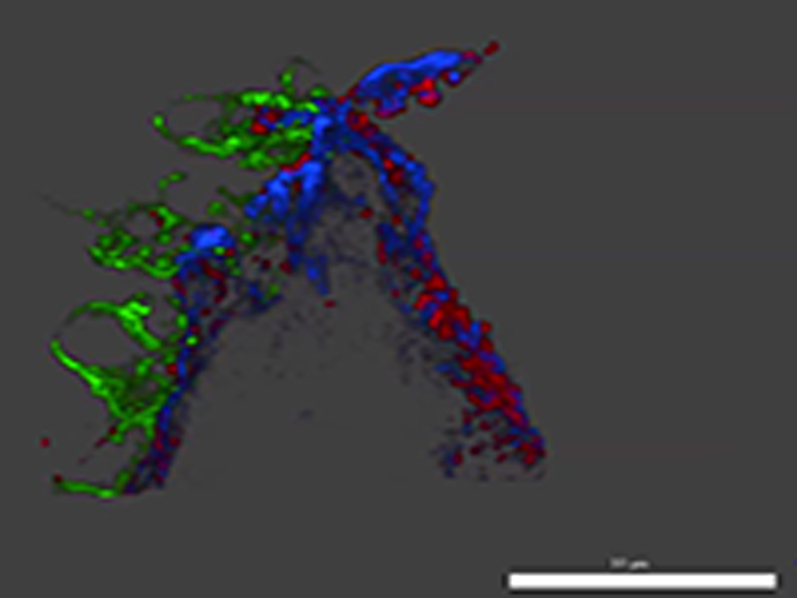
Online Video 8

Inhibition of the platelet P2Y_12_ receptor by cangrelor and thromboxane A_2_ (TxA_2_) formation by aspirin inhibited residual platelet aggregate formation in the presence of GPVI-Fc at low shear rate flow (Figure 5). When blood was treated with low, threshold-inhibitory concentrations of 5C4, results similar to those with GPVI-Fc at low shear rate were seen (Online Figure 7, Online Video 9). Platelets adhered to sites downstream of plaque fragments that recruited nearby flowing platelets into aggregates. Additional inhibition of the P2Y_12_ receptor and TxA_2_ formation significantly reduced residual platelet aggregate formation in the presence of low 5C4 concentrations (Online Figure 7).**Figure 5:**
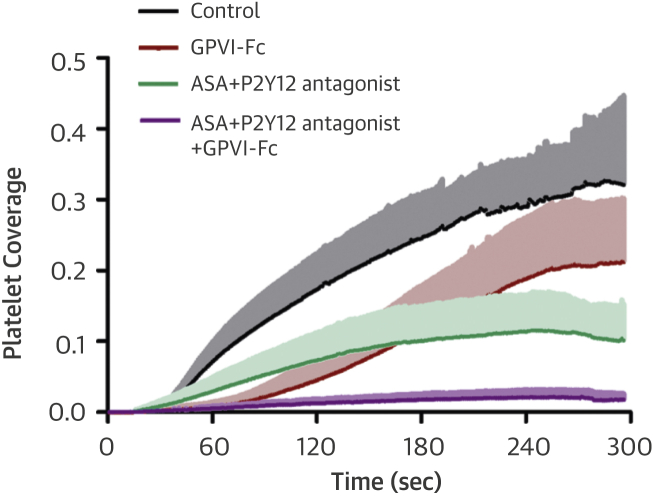
Residual Platelet Aggregate Formation in the Presence of GPVI-Fc Is Inhibited by Blockade of Platelet Cyclooxygenase and the P2Y_12_ Receptor Blood was pre-incubated for 5 min with buffer (control), GPVI-Fc (50 μg/ml), the P2Y_12_ receptor antagonist AR-C69931 (1 μM) added to blood containing acetylsalicylic acid (ASA) (1 mM), alone or in combination with GPVI-Fc. Blood was perfused over plaque at a shear rate of 550/s. Mean ± SD (n = 5). p < 0.005 for treatment with ASA + P2Y_12_ antagonist + GPVI-Fc (endpoint) compared with ASA + P2Y_12_ antagonist and GPVI-Fc. Abbreviations as in Figure 1.

**Figure:**
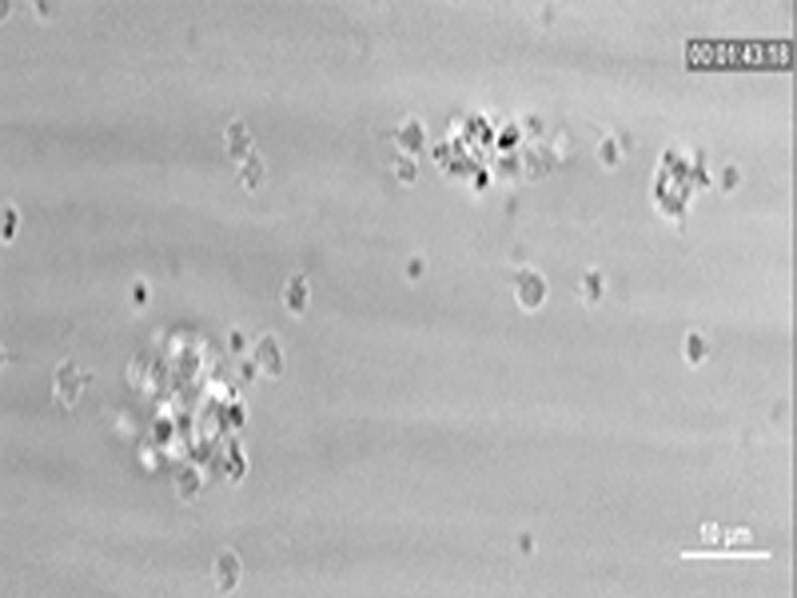
Online Video 9

## Discussion

The crucial role of GPVI-collagen interaction in plaque rupture- or erosion-triggered thrombotic occlusion stimulated the generation of specific inhibitors promising benefit beyond established dual-antiplatelet therapy. To help define the most effective and safest anti-GPVI strategy, we compared compounds targeting GPVI-collagen interaction in various tests of platelet response to plaque material under static and arterial flow conditions. We found compound-specific differences relevant to clinical trial design.

The antibodies recognizing platelet monomeric and dimeric GPVI (BLO8-1, 5C4) inhibited collagen- and plaque-induced platelet aggregation almost completely under static and flow conditions at low and high shear rates. Although preserved, platelet adhesion was transient rather than stable. Inhibition was less with GPVI-Fc. Dimeric GPVI is essential for binding to collagen and activation of platelets 16, 17, 18, and GPVI-Fc was constructed to mimic dimeric GPVI. It binds to collagen with high affinity, thus concealing GPVI-binding sites from platelets. Our results of collagen- and plaque-induced static platelet aggregation, in which the integrin α_2_β_1_- and the vWF-binding sites of collagen do not play a role in platelet activation, indicate that not all tandem GPO motifs on collagen were occupied by GPVI-Fc, even after pre-incubation with very high GPVI-Fc concentrations. These few unoccupied GPO sites may suffice to trigger GPVI-induced signaling and subsequent ADP and TxA_2_ release, which then autoamplifies platelet activation under static and low shear rate flow conditions. Indeed, we have previously shown that in static assays plaque-induced aggregation is largely dependent on dense granule secretion that is abolished by a combination of platelet cyclooxygenase inhibition and P2Y_12_-receptor blockade 26, 27.

How does soluble dimeric GPVI-Fc differ from platelet GPVI dimer? Plasmon surface resonance yielded a low dissociation constant of collagen GPVI-Fc binding (K_D_ = 1.17 × 10^−7^ M), as reported previously (17). In line with the high affinity of GPVI-Fc to collagen, fluorescent-labeled GPVI-Fc added to flowing blood rapidly bound to plaque. This binding was stable, indicating a very low off rate of GPVI-Fc. Differences between soluble GPVI-Fc and platelet GPVI dimer must be due to factors other than affinity. First, GPVI dimers are recruited from monomers during platelet activation and cluster in lipid rafts leading to a high local GPVI-dimer density on the platelet surface 15, 16, 31. Second, spacing of the 2 collagen binding sites on dimeric GPVI-Fc lacking the mucin-like stems of the platelet GPVI dimer might be less flexible and may not always fit with the GPO repeats exposed on plaque collagen (Central Illustration A, left). This might explain why even pre-incubation of plaque with very high GPVI-Fc concentrations did not enhance platelet inhibition under static and low shear rate flow conditions (Figure 1, Online Figures 1 and 4). Third, the superior suppression of plaque-induced platelet aggregation by anti-GPVI antibodies recognizing both monomeric and dimeric platelet GPVI suggests that monomeric GPVI is functionally important. By also binding to monomers, these antibodies might impair dimer recruitment during platelet activation 15, 16 (Central Illustration B).

**Central Illustration:**
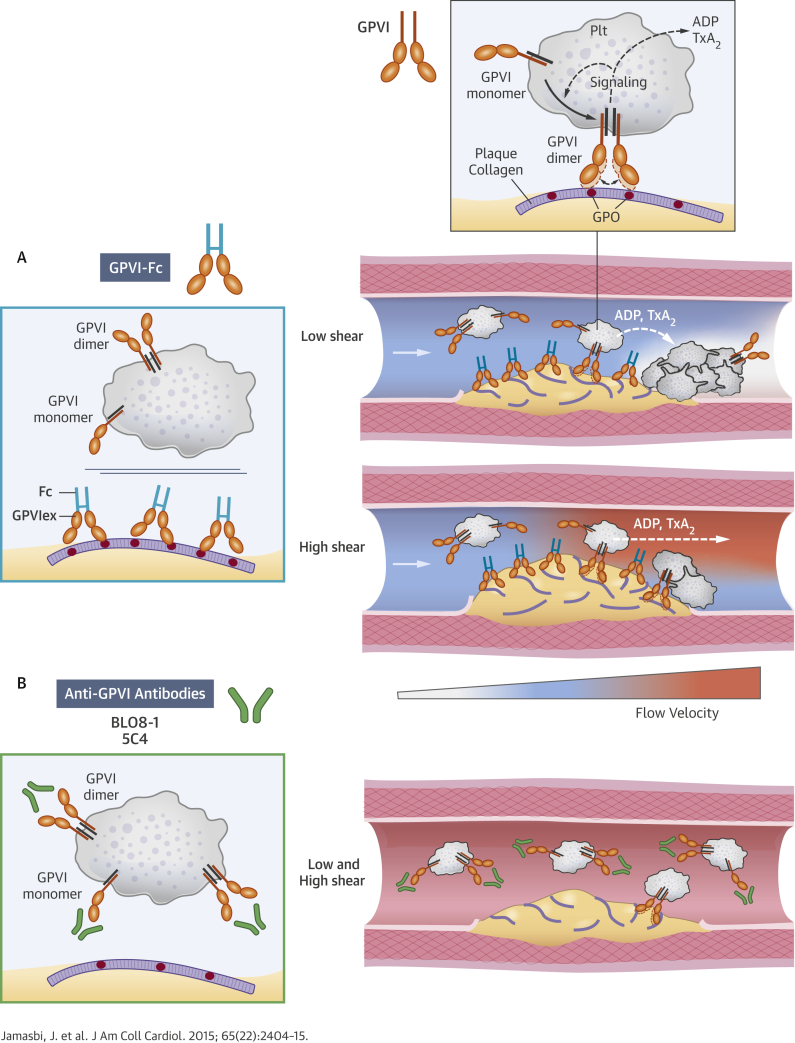
Differential Inhibition of Plaque-Induced Platelet Aggregate Formation by GPVI-Fc and Anti-GPVI Antibodies Glycoprotein VI is an essential platelet collagen receptor. **(A)** Dimeric fusion protein GPVI-Fc binds to exposed GPO sites of collagen up- and downstream after plaque rupture and competes with platelet GPVI dimer. Only a few GPO sites unoccupied by GPVI-Fc are needed to induce efficient platelet GPVI signaling with subsequent ADP and TxA_2_ release **(broken white curved arrow)** mediating stable platelet adhesion and aggregation in flow niches under low shear **(top)**, but not under high shear at sites of high-risk stenotic lesion ruptures as ADP and TxA_2_ are flushed away **(broken white arrow)** due to the high-flow velocity **(bottom)**; **white arrow** = direction of blood flow. **(B)** Anti-GPVI antibodies bind to platelets in the circulation and inhibit platelet aggregation after plaque rupture independently of flow and stenosis. ADP = adenosine diphosphate; Fc = fragment crystallizable region of IgG; GPO = glycine-proline-hydroxyproline; GPVI = glycoprotein VI; GPVIex = external domain of GPVI; Plt = platelet; TxA_2_ = thromboxane A_2_.

Our imaging studies demonstrate that GPVI-Fc bound equally well to plaque collagen at high and low shear rates, both up- and downstream of plaque fragments. However, plaque collagen sites not occupied by GPVI-Fc arrested platelets mainly under low shear rate flow, when flow niches downstream of plaque fragments might create nearly static conditions. On the basis of our imaging studies and ADP/TxA_2_ inhibition experiments, we suggest that GPVI signaling in a few platelets attaching downstream of the plaque induces stable adhesion and recruitment of circulating platelets into aggregates by the local release of TxA_2_ and ADP (Central Illustration A, top). Our results with low threshold-inhibitory concentrations of 5C4 further support such a mechanism. At high flow velocities, ADP and TxA_2_ will be flushed away, contributing to the increased effectiveness of GPVI-Fc at higher shear rates (Central Illustration A, bottom). An additional mechanism may be GPVI-Fc occupying vWF binding sites of collagen, which would inhibit vWF-mediated platelet adhesion relevant at high shear (32).

Surprisingly, the subtle (<8 μm) roughness of the plaque surface in our model created local differences in dynamics and extent of plaque-induced platelet aggregate formation at low and high shear rates in the presence of GPVI-Fc. Coronary thrombosis mostly arises from rupture of thin-capped (<65 μm) fibroatheroma, exposing the plaque necrotic core containing collagenous structures to circulating blood 6, 33, 34. This creates a new thrombogenic and rough luminal surface that will influence the dynamics of platelet adhesion and aggregation, probably similar to our ex vivo plaque model. Although nonstenotic coronary lesions with physiological wall shear rates (500/s to 600/s) may rupture, coronary atheromas with the highest risk to cause myocardial infarction are at least 50% stenotic with shear rates of ∼1,500/s (35). Rupture frequently occurs at the inflamed macrophage-rich plaque shoulder or at the most stenotic part, where endothelial shear stress is highest (36). The potent platelet inhibition by GPVI-Fc at high shear suggests locally enhanced antithrombotic efficacy of GPVI-Fc at ruptured high-risk lesions.

Given its action at the ruptured plaque and its flow-dependent platelet inhibition, GPVI-Fc is expected to be safer than GPVI antibodies for systemic bleeding risk. GPVI antibodies target all circulating platelets as do established antiplatelet drugs such as aspirin and P2Y_12_ antagonists, which increase bleeding. In fact, GPVI-Fc alone showed no increased bleeding in a phase I study (21). Under static and low shear rate flow conditions, GPVI-Fc, unlike GPVI antibodies, inhibited plaque- and collagen-initiated platelet aggregation only moderately. Because major bleeding complications arise mainly from vessels with lower flow velocities and shear rates than coronary arteries, safety concerns of triple platelet inhibition would appear lower with GPVI-Fc than with GPVI antibodies. Indeed, in mice, GPVI-Fc did not worsen bleeding time when combined with established common antithrombotic drugs such as aspirin, P2Y_12_ antagonists, and heparin alone or in combination (37).

Anti-GPVI antibodies causing systemic platelet inhibition could increase bleeding. Patients with rare genetic or acquired GPVI defects show a variable bleeding diathesis depending on clinical background (14). Although the tail bleeding time of GPVI-deficient mice is only moderately increased (21), depletion of platelet GPVI by antibody treatment severely compromised hemostasis in mice with concomitant aspirin therapy (38). Additionally, anti-GPVI antibodies can cause immune responses and long-lasting platelet GPVI depletion through GPVI shedding or other mechanisms (14). It is not yet known whether BLO8-1 and 5C4 have these effects. BLO8-1 is a human domain antibody (the smallest functional binding units of human Igs) that is less likely to be immunogenic and more likely to resist proteolytic degradation.

### Study limitations

This was an ex vivo study exposing plaque material to blood of healthy persons and not an in vivo study after plaque rupture of cardiovascular patients.

## Conclusions

Antibodies targeting platelet dimeric and monomeric GPVI are more effective inhibitors of plaque-induced platelet aggregation in static and dynamic models than GPVI-Fc masking GPVI-binding motifs on exposed plaque collagen. However, GPVI-Fc inhibition is flow dependent and increases with shear rate. This suggests a focused antithrombotic efficacy at rupture sites of high-risk lesions. Under low shear rate flow, GPVI-Fc synergizes with aspirin and P2Y_12_ antagonists in platelet inhibition. Thus, compounds targeting the GPVI-collagen axis have antiatherothrombotic potential beyond standard dual-antiplatelet therapy, and GPVI-Fc might be safer than antibodies directed against GPVI.

Perspectives **COMPETENCY IN MEDICAL KNOWLEDGE:** In static and flow models of human plaque-induced platelet activation, antibodies against platelet GPVI receptors are more potent inhibitors than a GPVI-Fc fusion protein that conceals plaque collagen from platelets. However, the potency of GPVI-Fc increases with shear rate and localizes the action to the site of plaque rupture or injury while preserving systemic platelet function, even after excess and prolonged exposure.

**TRANSLATIONAL OUTLOOK:** The differential actions of these compounds targeting the GPVI-collagen axis have implications for the design of clinical trials: anti–GPIV-antibodies might provide superior antithrombotic efficacy, but might be burdened with a higher bleeding risk than GPVI-Fc, especially when tested in combination with established dual-antiplatelet therapy.
