# Supplementary material for: Differential Inhibition of Human Atherosclerotic Plaque–Induced Platelet Activation by Dimeric GPVI-Fc and Anti-GPVI Antibodies: Functional and Imaging Studies
Source: J Am Coll Cardiol. 2015 Jun 9;65(22):2404–15. doi: 10.1016/j.jacc.2015.03.573 (PMC4452546; doi:10.1016/j.jacc.2015.03.573)
Supplement: Online Data [file mmc1.docx]

**ONLINE APPENDIX**

**METHODS**

**Reagents and antibodies**

Abciximab (ReoPro^®^) was obtained from Janssen Biologics B.V. (Leiden, the Netherlands). Adenosine 3-phosphate 5-phosphate (ADP) was purchased from Biopool (Wicklow, Ireland). DiOC6, Alexa Fluor®-405 goat anti-rabbit IgG, Zenon® Alexa Fluor®-405 and Zenon^®^ Alexa Fluor^®^-594 rabbit IgG labeling kits were from Life Technologies (Eugene, Oregon,). Goat anti-human IgG-Fc (gamma)-specific antibody conjugated with PE was obtained from eBioscience (San Diego, California). Goat anti-human IgG-Fc-specific antibody conjugated with Alexa Fluor^®^-594 came from Dianova (Hamburg, Germany). Anti- collagen type I and III antibodies (rabbit) purified by immunoaffinity chromatography were obtained from Rockland (Limerick, Pennsylvania). Human serum albumin, glycine, paraformaldehyde, acetylsalicylic acid (ASA) and mepacrine came from Sigma-Aldrich (Steinheim, Germany). Collagen (Horm) was obtained from Takeda (Linz, Austria). Anti- human CD41 antibody FITC conjugate was purchased from Invitrogen (Frederick, Maryland). EDTA came from Carl Roth GmbH (Karlsruhe, Germany). Recombinant lepirudin (Refludan^®^) was obtained from Celgene (Windsor, United Kingdom). TRIS was purchased from Biomol (Hamburg, Germany). The peptides TRAP (SFLLRN; thrombin-receptor activating peptide which binds to PAR-1) and RGDS were obtained from Bachem AG (Bubendorf, Switzerland). BLO8-1 (human single domain antibody against GPVI receptor) was from GlaxoSmithKline (Brentford, Middlesex, United Kingdom). Revacept^®^ (RP-15-260805, a dimeric GPVI-Fc fusion protein), Fc of human IgG1, and 5C4 (Fab-fragment of a monoclonal GPVI-blocking rat IgG1) were from AdvanceCOR GmbH (Martinsried, Germany). Rat IgG1 kappa isotype control antibody, and anti-mouse antibody conjugated with Dylight^®^ 488 was purchased from Abcam (Cambridge, United Kingdom). Monoclonal antibody against the GPVI dimer (m-Fab-F) was produced as previously described (1). AR-C69931MX (N6–2-(methylthioethyl)-2-(3,3,3-trifluoropropylthio)-β,γ-dichloromethylene ATP) was generously provided by Astra-Zeneca R&D Charnwood (Loughborough, United Kingdom). AR-C126532XX (1S,2S,3R,5S)-3-[7-[[(1R,2S)-2-(3,4-difluorophenyl)cyclopropyl]amino]-5-(propylthio)-3H-1,2,3-triazolo[4,5-d]pyrimidin-3-yl]-5-(2-hydroxyethoxy)-1,2-cyclopentanediol was from Cayman Chemicals (Ann Arbor, Michigan). Fluoro-Gel antifading reagent was from Electron Microscopy Sciences (Hatfield, Pennsylvania).

**Blood collection and platelet aggregation**

Hirudin (~200 U/ml; 13 μg/ml) -anticoagulated blood was obtained from healthy volunteers as described (2). Informed consent was obtained as approved by the Ethics Committee of the Faculty of Medicine of the University of Munich.

Platelet aggregation in blood was determined by multiple electrode aggregometry (MEA) using the Multiplate® device as described previously (3) according to a recently modified protocol (4). Blood samples were pre-incubated with the GPVI antibodies, GPVI-Fc or controls (see below) at 37°C for 3 min in the absence of stirring (4). Collagen (0.5µg/ml), plaque homogenate (833µg/ml), ADP (5µM), or TRAP (15µM) was added, stirring was started and the increase in electrical impedance was recorded continuously for 5 or 10 min. The mean value of two independent determinations is expressed in arbitrary “aggregation units” over the time period (AU*min) (cumulative aggregation values). Values of collagen- and plaque-induced platelet aggregation measured after 5 min were 343 ± 145 AU*min (mean ± SD, n=24), and 238 ± 71 AU*min (mean ± SD, n=29), respectively.

GPVI-Fc (109, 219, 437, 875, 1750 µg/ml) was also pre-incubated directly with plaque for 3 min at room temperature before transfer to the MEA cuvettes. As controls, we used equimolar concentrations of human Fc and rat IgG1 for GPVI-Fc and 5C4, respectively. Buffer only (0.1M TRIS, 0.1M glycine, pH 7.5) was used as control for BLO8-1.

**Analysis of platelet adhesion and thrombus formation in flowing whole blood**

For flow experiments, glass cover slips (Menzel, 24x60mm, # 1.5) coated with pooled plaque homogenates (1:20 dilution in PBS) were mounted into parallel plate flow chambers using sticky slides (0.1 Luer and 0.2 Luer sticky slides, ibidi^®^, Martinsried, Germany) which had been blocked with human serum albumin (4% in PBS). The flow chamber was then mounted on the stage of a fluorescence microscope (TE2000-E, Nikon) equipped with an incubation chamber (37°C). The plaque coated flow chambers were perfused with PBS followed by 4% human serum albumin (HSA) in PBS to block the glass coverslips.

Human whole blood was pre-incubated with mepacrine (10µM) or DiOC6 (1µM) (as specified in the Figure legends) for 5 min for platelet staining, and for further 5 min with GPVI-Fc, BLO8-1, 5C4, or control proteins. As none of the control proteins influenced platelet coverage, concurrent controls were run with untreated blood. Blood was perfused through the flow chamber at shear rates of 550/s, or 1100/s or 1500/s via a withdrawal syringe pump (Harvard Apparatus, Holliston, Massachusetts, USA). Fluorescence microscopy (Lambda DG4, Sutter Instruments; excitation: 485/25nm, emission: 528/38 nm) was performed for real time measurement of platelet adhesion and aggregate formation using a 10x air objective (NA 0.4) and a CoolSNAP HQ2 CCD camera (Photometrics). Fluorescence images were continuously recorded (1frame/sec) and analyzed by quantifying the binary fluorescent area fraction (1.0=total area) using the NIS-element 3.2 (Nikon) software package. The area visualized in this setting was 669µm x 896µm. Experiments were performed in duplicates. Values are the mean ± SD (measured each sec) of *n* experiments with blood from different donors.

**Imaging of GPVI-Fc and platelet binding to plaque homogenates in flow experiments**

Plaque–coated glass coverslips were mounted into parallel plate flow chambers as described above. Platelets were labelled by incubation of DiOC6 (1µM, 10min at 37°C) with blood. GPVI-Fc (100µg/40µl) was labeled with anti-humanFc-antibody conjugated with PE (0.5µg) or with Alexa Fluor594 (10µg) for 10min at room temperature, and added to blood at a final concentration of 50µg/ml before perfusion. Imaging was performed with two fluorescence microscopes:

(a) a Nikon TE2000-E microscope for measurement of binding of DiOC6-labelled platelets (excitation: 485/25 nm) and GPVI-Fc labelled with PE (excitation: 560/25nm) with the emission filter Quad-set F66-888 (DAPI/FITC/Cy3/Cy5) using a 10x objective, and for imaging platelets by differential interference contrast microscopy (DIC) in relation to binding of PE-labelled GPVI-Fc using a 100x oil objective (NA1.4) (1frame/2sec).

(b) a Leica SP5IIMP two-photon laser scanning laser microscope (TPSLM) equipped with a Ti: Sa pulsed laser (Spectra Physics MaiTai DeepSee) set at 770nm and a 63x oil objective (NA 1.40) for three dimensional imaging of platelet binding to plaque over time using fluorescence signal derived from autofluorescence of plaque components (410-490nm), platelets (DiOC6: 510-532nm), and GPVI-Fc labeled with anti-human Fc antibody conjugated with Alexa Fluor594 (601-634nm). Spectral settings were chosen to maximize detection efficiency while avoiding bleed through between the channels. Additional optical zoom was applied to further improve the overall resolution. Three dimensional image acquisition rate was 0.08-0.15Hz, dependent on the z-step size and z-depth of the acquired volume (single xy-frame rate; 1-2Hz). For 3D rendering the Huygens Professional 14.10 software package (Scientific Volume Imaging, Hilversum, the Netherlands) or the Leica LasX software (Leica microsystems GMBH, Mannheim, Germany) was applied.

For structured illumination microscopy (SIM) imaging of plaque collagen, GPVI-Fc and platelets, plaque homogenate -coated glass cover-slips were blocked with 4% HSA in PBS, incubated with anti-collagen type I and type III antibodies (100ng of each per sample; 15min at room temperature, wet chamber), and after washing (0.5% HSA in PBS) stained with AlexaFluor405-conjugated 2^nd^ Ab (200ng per sample; 10min at RT, wet chamber). After washing, the collagen-stained plaque homogenates were mounted into parallel plate flow chambers as described above. Blood was pre-incubated for 10min at 37°C with abciximab (20µg/ml) to block platelet aggregation, and GPVI-Fc labeled with anti-humanFc AlexaFluor594 antibody was added to blood just before start of perfusion. At 4 min after start of blood flow, samples were fixed by perfusion with 4% PFA in PBS containing 5mM EDTA for 5 min. Platelets were stained with an anti-CD41 antibody (dilution 1:200 in washing buffer; 2min) and subsequently with DyLight® 488-conjugated 2^nd^ Ab (dilution 1:100; 2min). After fixation and between the staining steps, the samples were perfused (350µl/min) for 3 min with washing buffer (0.5% HSA in PBS) containing 6.5µg/ml lepirudin. The flow chambers were filled with Fluoro-Gel, and samples were documented and analysed by SIM (5). Thin (0.2 μm) z-sections of high-resolution images were collected in three rotations for each channel using an ELYRA PS.1 (Carl Zeiss MicroImaging) microscope. Images were reconstructed using ZEN software (Carl Zeiss MicroImaging) based on the structured illumination algorithm developed by Heintzmann and Cremer (6). For 3D rendering ImagePro Premier 3D v9.1 (Media Cybernetics Inc., Rockville, Maryland) was applied. To estimate the roughness of the plaque surface, the maximal height of the plaque fragments was measured by SIM. The values were 6.12±1.65 µm (mean±SD; n=6).

**Statistical analysis**

Results are shown as mean ± SD from experiments conducted with blood from different donors (n). In dose-response experiments, aggregation in the presence of the specific inhibitor (GPVI-Fc, BLO8-1, 5C4) was calculated as % of aggregation in the presence of equimolar amounts of the relevant control protein or buffer (Fc-control protein, TRIS-glycine buffer, IgG1, respectively) for each donor. At every concentration inhibitor and concurrent controls were compared by the two-tailed paired *t*-test (*: p<0.05, **: p<0.01 or ***: p<0.001) or by Mann–Whitney *U*-test (§§§: p<0.001) as appropriate. Multiple parallel experimental conditions were compared by ANOVA with secondary pair comparisons by Student-Newman-Keuls method. Flow experiments were analyzed by 3-way ANOVA using shear rate, inhibitor treatment and time as independent factors. Within factor secondary comparisons were made by Holm-Sidak method.

**References**

1. Jung SM, Tsuji K, Moroi M. Glycoprotein (GP) VI dimer as a major collagen-binding site of native platelets: direct evidence obtained with dimeric GPVI-specific Fabs. J Thromb Haemost 2009;7:1347-55.

2. Penz SM, Reininger AJ, Toth O, Deckmyn H, Brandl R, Siess W. Glycoprotein Ibalpha inhibition and ADP receptor antagonists, but not aspirin, reduce platelet thrombus formation in flowing blood exposed to atherosclerotic plaques. Thromb Haemost 2007;97:435-43.

3. Toth O, Calatzis A, Penz S, Losonczy H, Siess W. Multiple electrode aggregometry: a new device to measure platelet aggregation in whole blood. Thromb Haemost 2006;96:781-8.

4. Bampalis VG, Brantl SA, Siess W. Why and how to eliminate spontaneous platelet aggregation in blood measured by multiple electrode aggregometry. J Thromb Haemost 2012;10:1710-4.

5. Elia N, Ott C, Lippincott-Schwartz J. Incisive imaging and computation for cellular mysteries: lessons from abscission. Cell 2013;155:1220-31.

6. Heintzmann R, Cremer C. Axial tomographic confocal fluorescence microscopy. Journal of microscopy 2002;206:7-23.

**Online Figure 1**

**Pre-incubation of plaque with excess concentrations of GPVI-Fc does not further inhibit platelets under static conditions. Left,** Blood was incubated with solvent (control), equimolar concentrations of GPVI-Fc (50µg/ml), or Fc protein (17µg/ml) for 3 min before stimulation with plaque homogenate (833µg/ml) for 10 min. **Right,** Plaque was pre-incubated with solvent (control), equimolar concentrations of GPVI-Fc (1750 µg/ml) or Fc (595 µg/ml) for 30 min at 37°C before transferring aliquots to blood in the MEA cuvettes and measurement of aggregation for 10 min. The GPVI-Fc and Fc concentrations for pre-incubation were 35-fold higher than the blood concentrations in the left panel. The final concentrations in blood were the same as the blood concentrations in the left panel. Cumulative aggregation values (AU*min) measured from 0 to 10 min are shown. Mean + SD (n=5).

**
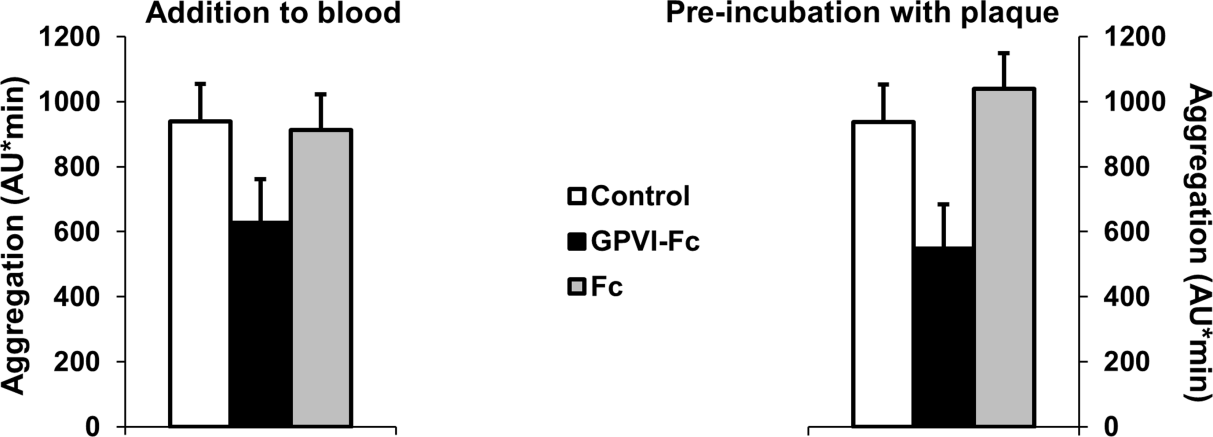
**

**Online Figure 2**

**BLO8-1 (A) and 5C4 (B) inhibit static platelet aggregation in blood stimulated by plaque, but not by ADP and TRAP (C).**

**Left**, Representative MEA tracings of plaque (833µg/ml) -induced platelet aggregation after pre-incubation with **(A)** BLO8-1 (10µg/ml) or **(B)** 5C4 (1.25µg/ml). Numbers show cumulative aggregation (AU*min) measured over 10 min. **Right**, Concentration-dependency of the effect of **(A)** BLO8-1 or **(B)** 5C4 on platelet aggregation measured over 5 min. Mean ± SD or median and box plot (in % of concurrent control) of (A) n=3-9 or (B) n=4-5 experiments. *: p < 0.05, **: p < 0.01, ***: p < 0.001 for comparison of treated to control platelets by two-tailed paired *t*-test or §§§: p<0.001 by Mann–Whitney *U*-test. **(C)** BLO8-1 (10µg/ml; left panel) or 5C4 (1.25µg/ml; right panel) do not affect platelet aggregation stimulated by ADP (5µM) or TRAP (15 µM) (mean ± SD, n=4).

**
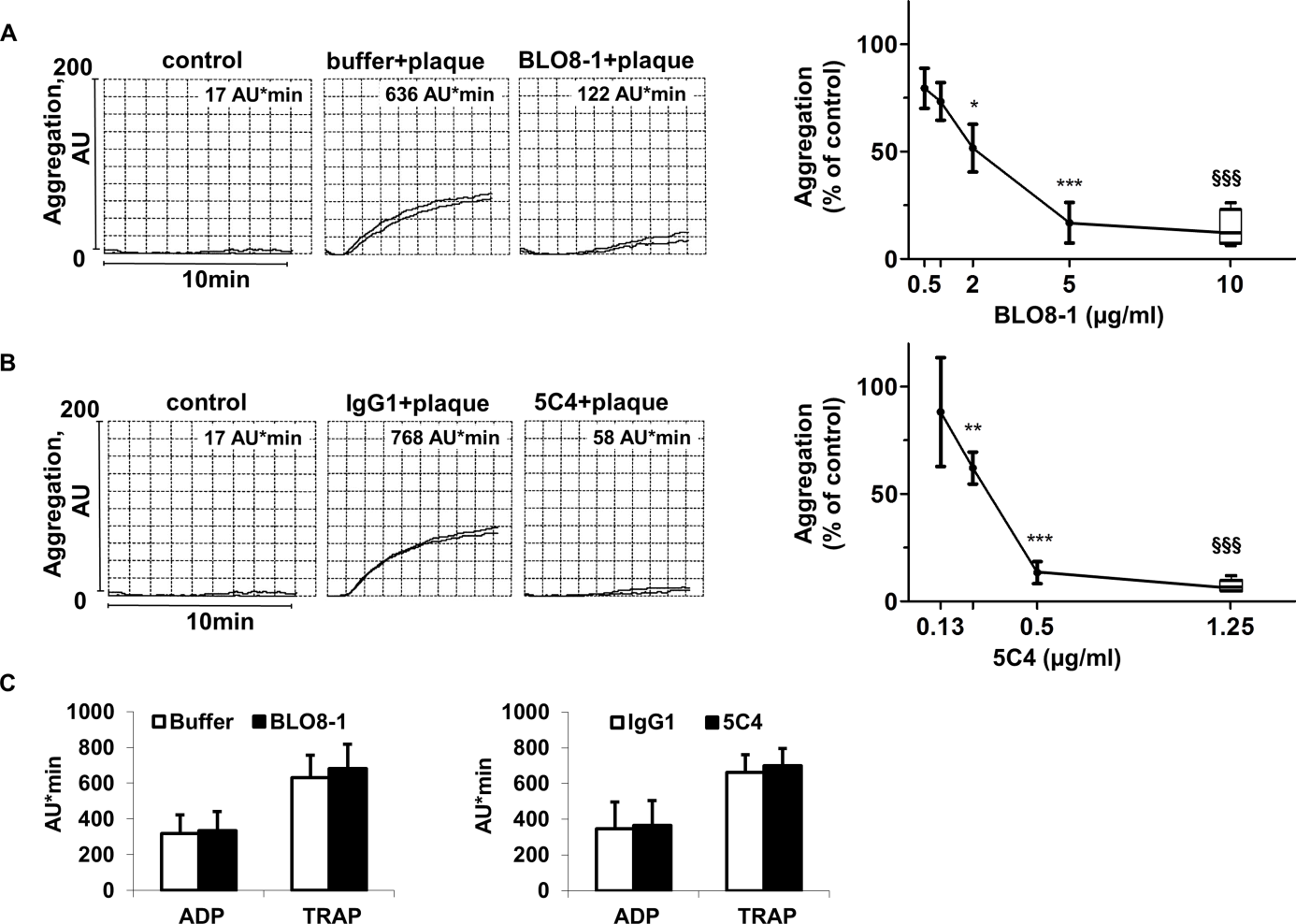
**

**Online Figure 3**

**Antibodies against monomeric and dimeric GPVI (5C4), dimeric GPVI only (m-Fab-F), and GPVI-Fc inhibit plaque stimulated static platelet aggregation to a different extent**

**(A)** Representative MEA tracings. Blood was pre-incubated with 5C4 (1.25µg/ml), m-Fab-F (100µg/ml) or GPVI-Fc (50µg/ml) before stimulation with plaque (208µg/ml) for 10 min. **(B)** Bar diagram of the influence of 5C4, m-Fab-F and GPVI-Fc on plaque-induced platelet aggregation measured over 10 min. Mean ± SD (n=5); * p < 0.05, ** p < 0.01, *** p < 0.001 by ANOVA and secondary pair comparisons by Student-Newman-Keuls method as indicated.

**
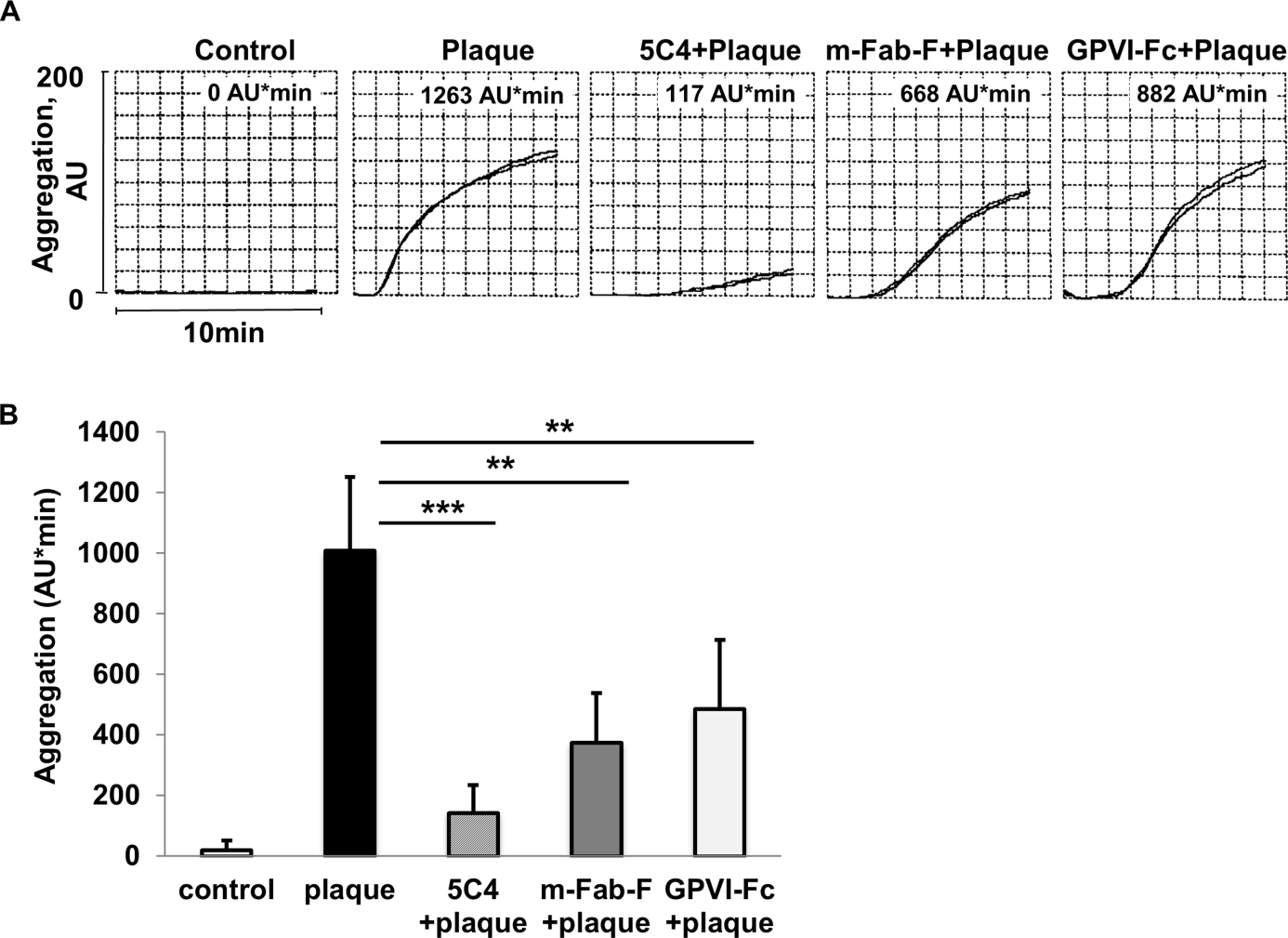
**

**Online Figure 4**

**Pre-incubation of plaque with excess concentrations of GPVI-Fc does not further inhibit platelets under low shear rate flow conditions.** Blood was pre-incubated with DiOC6 for 10min for platelet visualisation. **Left** Solvent (control), equimolar concentrations of Fc protein (17µg/ml) or GPVI-Fc (50µg/ml), were added to blood before perfusion over plaque-coated coverslips. **Right,** Plaque-coated coverslips were pre-incubated with solvent (control), or high equimolar concentrations of Fc (34µg in 40µl = 850µg/ml) or GPVI-Fc (100µg in 40µl = 2500µg/ml) for 5 min at 37°C before perfusion with blood (shear rate 550/s). The concentrations for pre-incubation were 50-fold higher than the blood concentrations in the left panel. Mean ± SD (n= 4).

**
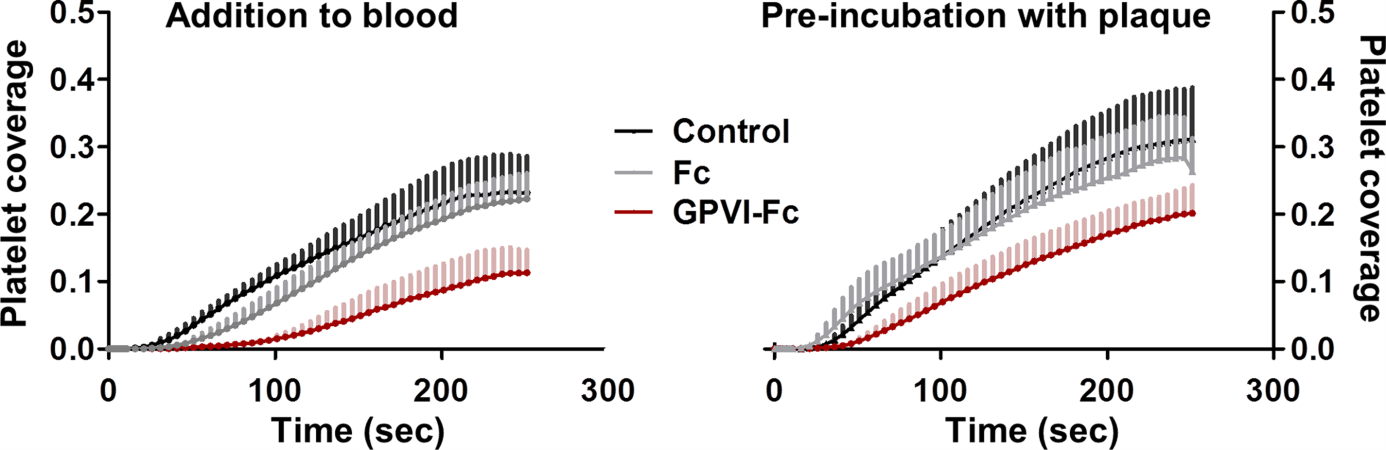
**

**Online Figure 5**

**Rapid and saturable GPVI-Fc binding to the more homogenous small plaque components under flow.**

GPVI-Fc (50µg/ml final concentration) labeled with PE-conjugated anti-human-Fc-antibody was added to blood before perfusion over plaque homogenate at 550/s. Fluorescence images were taken by video microscopy (1frame/2sec) using a 100x NA 1.4 oil objective (see suppl. video 4). Fields of view containing only the more homogenous small atherosclerotic plaque components were cropped and GPVI-Fc binding was quantified with the NIS-element 3.2 (Nikon) software. Values are % of maximal GPVI-Fc binding. Mean +SD (n=4).

**
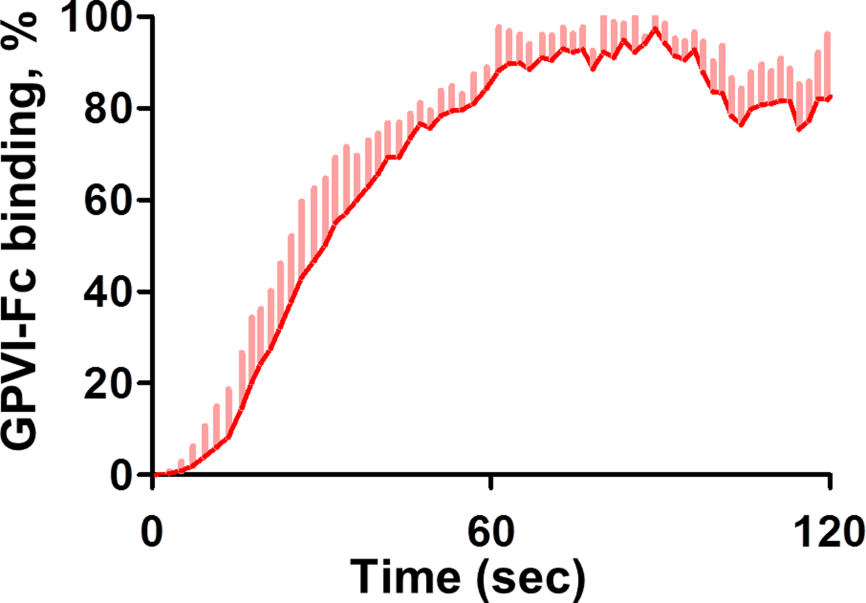
**

**Online Figure 6**

**Platelets (green) aggregate down-stream of plaque material (blue) under low, but not high shear flow in the presence of GPVI-Fc (red). Imaging by two-photon laser scanning microscopy (TPSLM).**

DiOC6 was added to blood for platelet labeling (green), and GPVI-Fc (50µg/ml final concentration) labeled with AlexaFluor594-conjugated anti-humanFc-antibody (red) was added to blood before perfusion over plaque. Representative TPSLM micrographs of platelet attachment to plaque (autofluorescence in blue) at different times after start of blood perfusion at (**A)** low shear rate (550/s), and **(B)** high shear rate (1500/s). Bar = 5 µm. Arrow, flow direction. See also suppl. video 7.

**
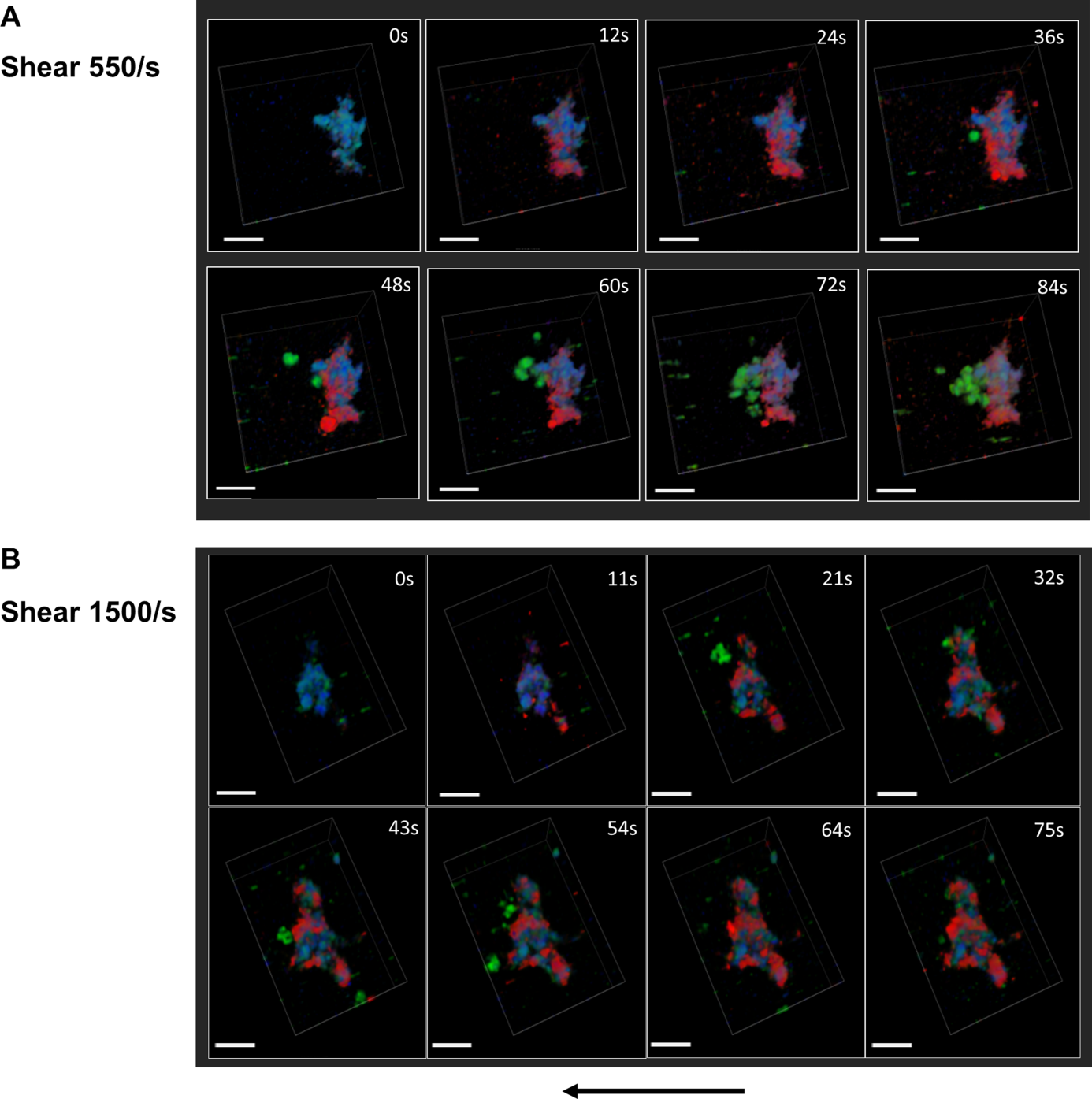
**

**Online Figure 7**

**Residual platelet aggregate formation in the presence of low 5C4 concentrations is inhibited by blockade of platelet cyclooxygenase and the P2Y_12_ receptor.**

Blood was pre-incubated for 5 min with buffer (control), a low threshold inhibitory concentration of 5C4 (0.5 µg/ml), the P2Y_12_ receptor antagonist AR-C126532XX (5µM) added to blood containing ASA (1mM), alone or in combination with 5C4. Blood was perfused over plaque at a shear rate of 550/s. Mean ± SD (n=6). p< 0.01 for treatment with ASA+P2Y_12_ antagonist+5C4 low (endpoint) compared to ASA+P2Y_12_ antagonist, and 5C4 low.

**
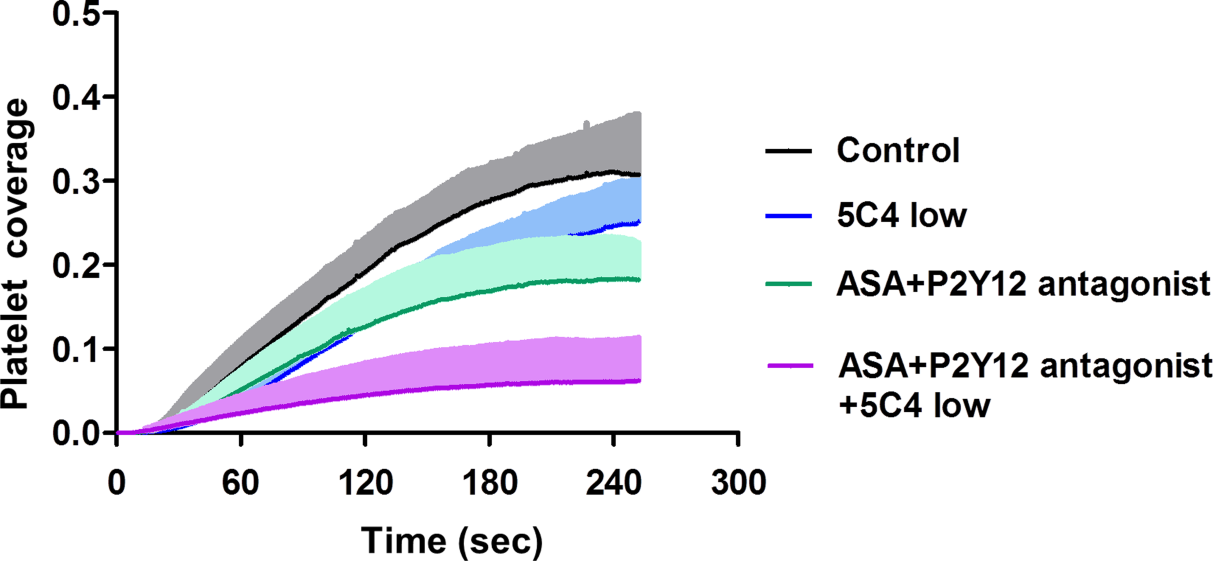
**

**Legends of Supplementary Videos**

**Video 1: Platelets aggregate onto plaque in flowing blood.** Platelets were stained with mepacrine, and blood was perfused (shear rate: 550/s) over plaque homogenate for 5 min. A 10x NA 0.4 air objective was used. Flow direction: from right to left.

**Video 2: Platelet aggregation onto plaque is delayed and reduced after treatment with GPVI-Fc**. Blood was treated with GPVI-Fc (50µg/ml) and perfused (shear rate: 550/s) over plaque homogenate for 5 min. Platelets were stained with mepacrine. Flow direction: from right to left.

**Video 3: Platelet aggregation onto plaque is inhibited after treatment with anti-GPVI antibodies. Platelet adhesion is more transient than stable.** Blood was treated with 5C4 (1.25µg/ml) and perfused (shear rate: 550/s) over plaque homogenate for 5 min. Platelets in blood were stained with mepacrine. Flow direction: from right to left.

**Video 4: GPVI-Fc binds rapidly to small and large plaque fragments**

GPVI-Fc labeled with PE-conjugated anti-humanFc-antibody was added to blood (final concentration 50µg/ml) just before start of perfusion (shear rate: 550/s) over plaque homogenate. GPVI-Fc was detected by fluorescence microscopy using a 100x NA 1.4 oil objective. Flow direction: from right to left.

**Video 5: Dynamics of GPVI-Fc binding and platelet attachment onto plaque fragments.**

GPVI-Fc rapidly binds to small and large plaque fragments up- and downstream, platelets adhere in flow niches to large plaque fragments leading to platelet aggregate formation. GPVI-Fc labeled with PE-conjugated anti-humanFc-antibody was added to blood (final concentration 50µg/ml) just before start of perfusion (shear rate: 550/s) over plaque homogenate. Plaque fragments and platelets were detected by DIC, and PE-labelled GPVI-Fc was detected by fluorescence microscopy (frame/2s) using a 100x NA 1.4 oil objective. Flow direction: from right to left.

**Video 6: Regardless of rapid GPVI-Fc binding to plaque, platelets aggregate downstream of large plaque fragments at low shear rate flow.**

Three dimensional visualization (TPLSM) of platelet attachment (green) to autofluorescent plaque material (blue) in the presence of GPVI-Fc (red). Dataset is presented as an extended depth of field projection over time (total thickness 10µm; z-step = 0.7µm). Platelets were stained with DiOC6. GPVI-Fc labeled with AlexaFluor594 -conjugated anti-human-Fc-antibody was added to blood (final GPVI-Fc concentration 50µg/ml) just before start of perfusion (shear rate: 550/s) over plaque homogenate. Flow direction: from right to left.

**Video 7: Platelets adhere and aggregate to plaque fragments downstream at low shear in the presence of GPVI-Fc.**

Blood was treated with GPVI-Fc (50µg/ml) and perfused (shear rate: 550/s) over plaque homogenate. Plaque homogenate and platelets were visualised by DIC microscopy (1 frame/s) using a 100 x NA1.4 oil objective. Flow direction: from right to left.

**Video 8: Platelets (green) adhere downstream to discrete sites of plaque collagen (blue) not occupied by but in close proximity to GPVI-Fc (red) as revealed by SIM imaging**

Three-dimensional animation of the SIM image in Fig. 4. Total thickness, 3.6 µm; z-step, 0.2µm.

**Video 9: Platelets adhere and aggregate downstream of plaque fragments in the presence of low threshold- inhibitory concentrations of 5C4**

Blood was treated with 5C4 (0.5µg/ml) and perfused (shear rate: 550/s) over plaque homogenate. Plaque homogenate and platelets were visualised by DIC microscopy (1 frame/s) using a 100x NA 1.4 oil objective. Flow direction: from right to left.
